# Recent COVID-19 Vaccination and Risk of SARS-CoV-2 Transmission

**DOI:** 10.1001/jamanetworkopen.2026.12609

**Published:** 2026-05-15

**Authors:** Sara C. Benist, Sarah E. Smith-Jeffcoat, H. Keipp Talbot, Yuwei Zhu, Jonathan E. Schmitz, Theresa A. Scott, Melissa S. Stockwell, Son H. McLaren, Ellen D. Sano, Celibell Y. Vargas, Ana Valdez de Romero, Helen Y. Chu, Janet A. Englund, Erica Clark, Melissa P. MacMillan, Adam S. Lauring, E. Ivy Oyegun, J. Bradford Bertumen, Carlos G. Grijalva, Hannah L. Kirking

**Affiliations:** 1 National Center for Immunization and Respiratory Diseases, Centers for Disease Control and Prevention, Atlanta, Georgia; 2 Oak Ridge Institute for Science and Education, Oak Ridge, Tennessee; 3 Vanderbilt University Medical Center, Nashville, Tennessee; 4 Columbia University Irving Medical Center, New York, New York; 5 University of Washington, Seattle, Washington; 6 Department of Internal Medicine, University of Michigan, Ann Arbor

## Abstract

**Importance:**

COVID-19 vaccine effectiveness (VE) is typically studied in the context of reducing the risk of severe illness and death. Few studies have estimated VE in preventing transmission and infection with current levels of SARS-CoV-2 population immunity.

**Objective:**

To estimate COVID-19 VE against transmission and infection within households.

**Design, Setting, and Participants:**

This cohort study was a prospective, case-ascertained household transmission study (performed in New York, Tennessee, and Washington) in which the first household member with confirmed SARS-CoV-2 infection (primary case participant) was identified through outpatient settings and enrolled with their household contacts from January 1, 2024, to January 31, 2025. Participants provided demographic information, and COVID-19 vaccination history was verified by study staff. After enrollment, participants were instructed to collect daily nasal swabs for 10 days regardless of symptoms. Nasal swabs were tested for SARS-CoV-2 via reverse transcription–polymerase chain reaction.

**Exposure:**

COVID-19 vaccination history in primary case participants and household contacts categorized as time from most recent vaccination to COVID-19 onset in the primary case participant (≤6 months, 7-12 months, >12 months, and unvaccinated [reference group]).

**Main Outcomes and Measures:**

Household contacts were considered infected if at least 1 swab tested positive for SARS-CoV-2. Secondary infection risk was calculated as the number of infected contacts divided by the total number of contacts. Adjusted relative risk (ARR) of infection was estimated using a multivariable Poisson regression model, with generalized estimating equations accounting for household-level clustering. Vaccine effectiveness was calculated as 1 minus the ARR of the primary case participant and household contacts’ vaccination status to estimate VE against transmission and against infection, respectively.

**Results:**

This analysis included 362 primary case participants (median [IQR] age, 35 [10-53] years; 199 female [55.0%]) and 763 household contacts (median [IQR] age, 29 [12-44] years; 399 female [52.3%]). SARS-CoV-2 infection was detected in 476 household contacts during follow-up for a secondary infection risk of 62.4% (95% CI, 58.7%-65.5%). Household contacts of primary case participants vaccinated 6 months or less before onset had a lower infection risk compared with contacts of unvaccinated primary case participants (ARR, 0.57 [95% CI, 0.35-0.93]). There was no statistically significant difference in infection risk based on vaccination status of household contacts.

**Conclusions and Relevance:**

In this cohort study, recent COVID-19 vaccination was associated with a reduced risk of SARS-CoV-2 transmission. These findings suggest that COVID-19 vaccination may have an indirect benefit of decreasing transmission and thus reducing overall exposure to SARS-CoV-2.

## Key Points

**Question:**

How effective is COVID-19 vaccination in preventing transmission of SARS-CoV-2?

**Findings:**

In this cohort study of 362 primary case participants with SARS-CoV-2 infection and their 763 household contacts, 62% of household contacts were infected with SARS-CoV-2. Household contacts of primary case participants vaccinated 6 months or less before onset had nearly one-half the infection risk compared with contacts of unvaccinated primary case participants.

**Meaning:**

These findings suggest that COVID-19 vaccination may have an indirect benefit of decreasing transmission and thus reducing overall exposure to SARS-CoV-2.

## Teaser

This cohort study estimates COVID-19 vaccine effectiveness in preventing SARS-CoV-2 transmission and infection within US households.

## Introduction

SARS-CoV-2 infections continue to cause considerable morbidity and mortality in the US.^1^ COVID-19 vaccination has been shown to reduce severe COVID-19, including hospitalization and death.^2,3^ There are limited published data on how COVID-19 vaccination reduces the risk of infection or transmission, which are important factors in understanding how vaccines protect at-risk populations, reduce strain on health care systems, and reduce opportunities for new viral variants. Even a modest reduction in the risk of infection or transmission could have indirect benefits on community transmission and burden. Few studies have evaluated these questions with current levels of SARS-CoV-2 population immunity from both prior infections and vaccinations and recent circulating variants.^4^ Household transmission studies are specifically designed to address these epidemiologic questions in settings in which household members with different levels of SARS-CoV-2 immunity closely intermingle.^5^ The objective of this investigation was to estimate COVID-19 vaccine effectiveness (VE) against transmission (VEt) and infection (VEi).

## Methods

### Study Design

This cohort study included households enrolled in the Respiratory Infection: Gauge of Household Transmission (RIGHT) study, a prospective, case-ascertained household transmission study conducted in New York, Tennessee, and Washington from January 1, 2024, to January 31, 2025. This study was reviewed and approved by the Vanderbilt University Medical Center Institutional Review Board (45 CFR part 46.114; 21 CFR part 56.114). All participants provided written informed consent. This study followed the Strengthening the Reporting of Observational Studies in Epidemiology (STROBE) reporting guideline for cohort studies.

Individuals with laboratory-confirmed SARS-CoV-2 infection (primary case participants) were enrolled at 6 days or less from illness onset along with their household contacts. A primary case participant was defined as the first individual in the household to experience the onset of symptoms. Households with more than 1 individual with the same onset date as the primary case participant were excluded. At enrollment, all participants completed surveys that collected demographic information (including age, sex, and race and ethnicity), COVID-19 vaccination history, and retrospective symptom diaries (which included in-home prevention behaviors). Self-reported race and ethnicity were asked as 2 separate questions (eMethods in Supplement 1) and were combined into the following categories for analysis: Asian, non-Hispanic; Black, non-Hispanic; Hispanic or Latino; White, non-Hispanic; other (including American Indian or Alaska Native, non-Hispanic; Middle Eastern or North African, non-Hispanic; Native Hawaiian or Pacific Islander, non-Hispanic; multiracial, non-Hispanic; other; or unknown). Race and ethnicity data were collected to help describe the participants included in the study. Participants also self-collected a blood specimen for SARS-CoV-2 anti-nucleocapsid (anti-N) antibody testing. Primary case participants may have received infection prevention information from their clinicians; no infection prevention information was provided as part of participation in this investigation. Starting at enrollment, participants completed daily prospective symptom diaries (including in-home prevention behaviors) and collected daily nasal swabs for 10 days. Nasal swabs were tested for SARS-CoV-2 via reverse transcription–polymerase chain reaction, regardless of symptoms. Whole-genome sequencing was performed on the polymerase chain reaction–positive nasal swab with the lowest cycle threshold value for each participant. The eMethods in Supplement 1 provide a full description of the study design.

Participants included in this analysis had at least 5 daily nasal specimens with valid results, COVID-19 vaccination information, and a single primary case participant in their household. Participants were excluded if they received a COVID-19 vaccination 14 days or less prior to illness onset in the primary case participant.

### Defining SARS-CoV-2 Infection and Vaccination Status

Household contacts were considered to have SARS-CoV-2 infection if at least 1 nasal swab tested positive for SARS-CoV-2 regardless of symptoms. COVID-19 vaccination status for primary case participants and household contacts was based on the most recent verified COVID-19 vaccine dose (eMethods in Supplement 1). Any COVID-19 vaccine manufacturer and formulation were included. Vaccination status was categorized by time from most recent COVID-19 vaccination to associated primary case participant symptom onset (≤6 months, 7-12 months, >12 months, and unvaccinated).

### Statistical Analysis

We used a multivariable Poisson regression model, with generalized estimating equations accounting for household-level clustering, to estimate the adjusted relative risk (ARR) of SARS-CoV-2 infection among household contacts by primary case participant vaccination status (reference group, unvaccinated primary case participants) and household contact vaccination status (reference group, unvaccinated household contacts) (eMethods in Supplement 1). The VEt and VEi were calculated as 1 minus the ARR of vaccination status of primary case participants and vaccination status of household contacts, respectively. A sensitivity analysis was performed among households in which all members had the same vaccination status to understand the combined effects (VEi and VEt) of the vaccine in reducing risk if both the primary case participant and household contacts were recently vaccinated. The data analysis was performed using R, version 4.5.0 (R Foundation for Statistical Computing).

## Results

### Participant Characteristics

**Figure 1.**
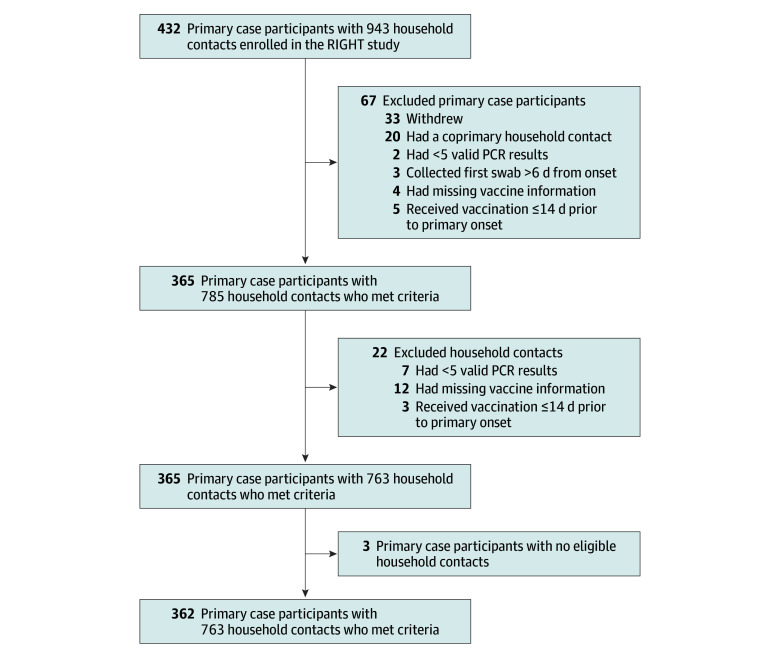
Flow Diagram of Participants Meeting Analysis Inclusion Criteria Among All Enrolled in the Respiratory Infection: Gauge of Household Transmission (RIGHT) SARS-CoV-2 Household Transmission Study PCR indicates polymerase chain reaction.

Of 432 households with a primary case participant with confirmed SARS-CoV-2 infection, 362 were eligible for this analysis (median [IQR] age, 35 [10-53] years; 199 female [55.0%], 155 male [42.8%], and 8 with unknown or missing sex [2.2%]; 14 identifying as Asian [3.9%], 38 as Black [10.5%], 100 as Hispanic or Latino [27.6%], 184 as White [50.8%], and 26 as other [7.2%] race and ethnicity) and included 763 household contacts (median [IQR] age, 29 [12-44] years; 399 female [52.3%], 348 male [45.6%], and 16 with unknown or missing sex [2.1%]; 23 identifying as Asian [3.0%], 81 as Black [10.6%], 278 as Hispanic or Latino [36.4%], 318 as White [41.7%], and 63 as other [8.3%] race and ethnicity) (Figure 1; Table 1). Peak enrollment occurred in August 2024. A total of 206 primary case participants (56.9%) reported at least 1 chronic health condition compared with 334 household contacts (43.8%). A total of 155 primary case participants (42.8%) and 330 household contacts (43.3%) had SARS-CoV-2 anti-N antibody detected.

**Table 1.** Characteristics of Participants Enrolled in the RIGHT SARS-CoV-2 Household Transmission Study Stratified by Primary Case vs Household Contact

Characteristic	Participants, No. (%)^a^		
	Overall (N = 1125)	Primary case (n = 362)	Household contact (n = 763)
Age at enrollment, y			
0-4	132 (11.7)	66 (18.2)	66 (8.7)
5-17	249 (22.1)	49 (13.5)	200 (26.2)
18-49	485 (43.1)	141 (39.0)	344 (45.1)
≥50	259 (23.0)	106 (29.3)	153 (20.1)
Sex			
Female	598 (53.2)	199 (55.0)	399 (52.3)
Male	503 (44.7)	155 (42.8)	348 (45.6)
Unknown or missing	24 (2.1)	8 (2.2)	16 (2.1)
Race and ethnicity			
Asian, non-Hispanic	37 (3.3)	14 (3.9)	23 (3.0)
Black, non-Hispanic	119 (10.6)	38 (10.5)	81 (10.6)
Hispanic or Latino	378 (33.6)	100 (27.6)	278 (36.4)
White, non-Hispanic	502 (44.6)	184 (50.8)	318 (41.7)
Other^b^	89 (7.9)	26 (7.2)	63 (8.3)
Chronic health conditions^c^			
≥1	540 (48.0)	206 (56.9)	334 (43.8)
0	577 (51.3)	154 (42.5)	423 (55.4)
Missing	8 (1.0)	2 (1.0)	6 (1.0)
Baseline SARS-CoV-2 anti-N detection^d^			
Detected or evidence of recent infection	485 (43.1)	155 (42.8)	330 (43.3)
Not detected or no evidence of recent infection	257 (22.8)	82 (22.6)	175 (22.9)
Unknown or no blood specimen collected <1 wk	383 (34.0)	125 (34.5)	258 (33.8)
Recent COVID-19 vaccination status^e^			
≤6 mo ago	104 (9.2)	35 (9.7)	69 (9.0)
7-12 mo ago	189 (16.8)	69 (19.1)	120 (15.7)
>12 mo ago	515 (45.8)	166 (45.9)	349 (45.7)
Unvaccinated	317 (28.2)	92 (25.4)	225 (29.9)
Enrollment state			
Tennessee	620 (55.1)	214 (59.1)	406 (53.2)
New York	309 (27.5)	82 (22.7)	227 (29.8)
Washington	196 (17.4)	66 (18.2)	130 (17.0)
Enrollment timing			
January 1 to June 30, 2024	502 (44.6)	157 (43.4)	345 (4.5)
July 1, 2024, to January 31, 2025	623 (55.4)	205 (56.6)	418 (54.8)
Total No. of people in home			
<4	NA	228 (63.0)	NA
≥4	NA	134 (37.0)	NA
Relationship to primary case participant			
Child or child-in-law	NA	NA	178 (23.3)
Parent or guardian, parent-in-law	NA	NA	215 (28.2)
Sibling	NA	NA	133 (17.4)
Spouse, partner, fiancé(e)	NA	NA	182 (23.9)
Other^f^	NA	NA	55 (7.2)
Isolated from others in the home during first 5 d of primary case participant’s onset^g^			
Yes	15 (1.3)	7 (1.9)	8 (1.0)
No	1110 (98.7)	355 (98.1)	755 (99.0)
Wore a mask in the home during first 5 d of primary case participant’s onset^h^			
Yes	49 (4.4)	25 (6.9)	24 (3.1)
No	1076 (95.6)	337 (93.1)	739 (96.9)

Abbreviations: Anti-N, anti-nucleocapsid; NA, not applicable; RIGHT, Respiratory Infection: Gauge of Household Transmission.

^a^ Participant type is defined as either being a primary case participant (ie, the first with confirmed SARS-CoV-2 infection or with the earliest onset of symptoms in the household) or a household contact.

^b^ Included American Indian or Alaska Native, non-Hispanic; Middle Eastern or North African, non-Hispanic; Native Hawaiian or Pacific Islander, non-Hispanic; multiracial, non-Hispanic; other; or unknown.

^c^ Chronic health conditions were self-reported and included the following: asthma, other nonasthma lung disease, cardiovascular or heart disease (including high blood pressure or hypertension), cancer, diabetes, HIV or immunosuppressing condition, obesity, kidney disease, liver disease, prematurity, cystic fibrosis, or other medical condition.

^d^ Evidence of recent SARS-CoV-2 infection was defined as having detectable SARS-CoV-2 anti-N antibodies in a blood specimen collected within 1 week of the primary case participant’s symptom onset. No evidence of recent SARS-CoV-2 infection was defined as not having detectable SARS-CoV-2 anti-N antibodies in a blood specimen collected within 1 week of the primary case participant’s symptom onset.

^e^ COVID-19 vaccination status was defined according to months from the most recent dose to the primary case participant’s symptom onset and grouped into the following categories: 6 months or less, 7 to 12 months, and more than 12 months. Participants who did not have at least 1 verified vaccine dose or self-reported at least 1 vaccine dose with a vaccination date and either a vaccine manufacturer or vaccination location were considered unvaccinated.

^f^ Included grandchild or great-grandchild (n = 1), grandparent or great-grandparent (n = 16), other relative (n = 21), or other nonrelative (n = 17).

^g^ Participant spent 15 minutes or less with other people in the home during the first 5 days of the primary case participant’s symptom onset.

^h^ Participant isolated from others or wore a mask when around others in the home during the first 5 days of the primary case participant’s symptom onset.

**Figure 2.**
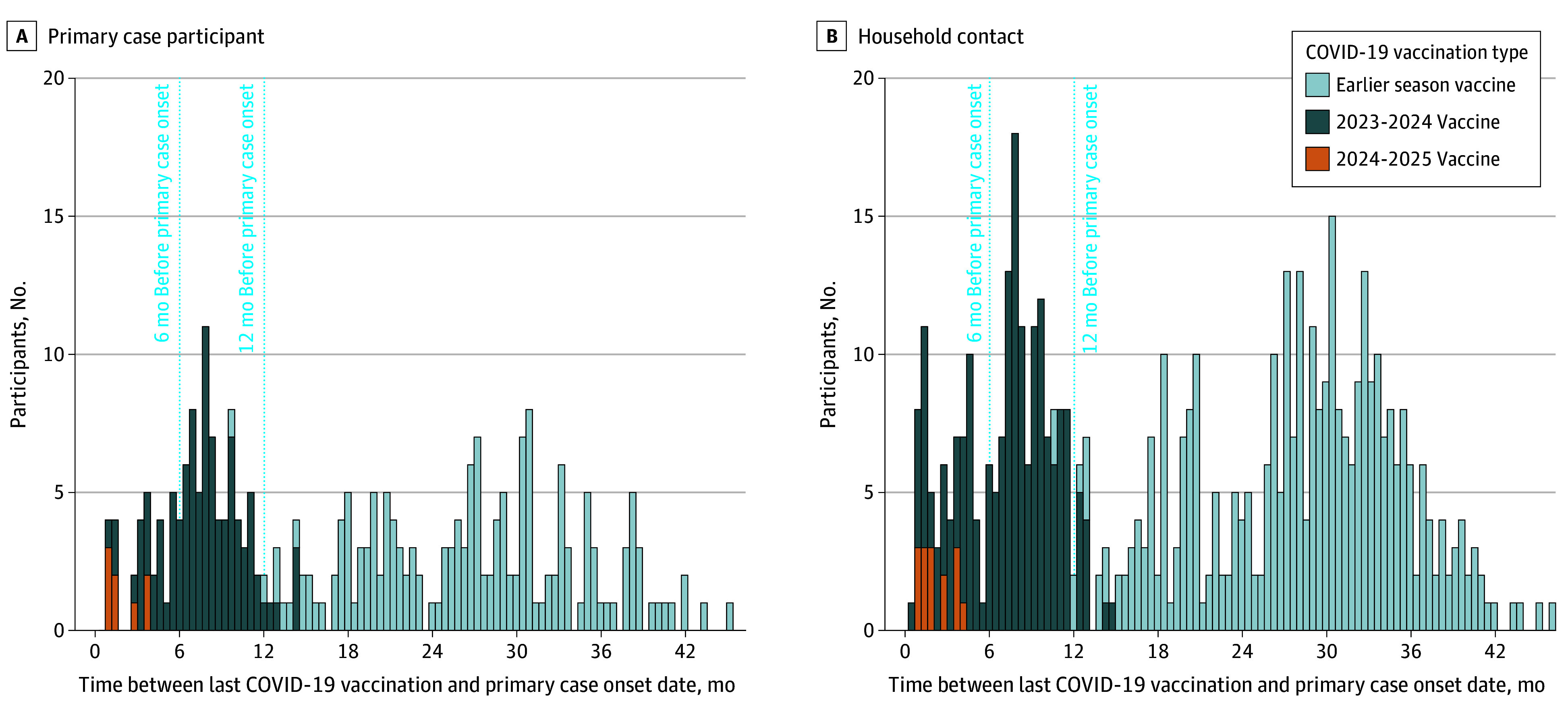
Histogram of Time From Most Recent COVID-19 Vaccination to Primary Case Participant Onset by Vaccine Type and Participant Type Vaccine type refers to the most recent COVID-19 vaccine received by the participant categorized as a 2024-2025 COVID-19 vaccine, a 2023-2024 COVID-19 vaccine, or a previous season prior to release of the 2023-2024 COVID-19 vaccines. Participant type is defined as either the first member of a household with a confirmed case of SARS-CoV-2 infection (primary case participant) or a household contact.

Primary case participants and household contacts had a similar distribution of COVID-19 vaccination status, with 35 (9.7%) and 69 (9.0%), respectively, having received a vaccine within 6 months of primary onset (7-12 months, 69 [19.1%] vs 120 [15.7%]; >12 months, 166 [45.9%] vs 349 [45.7%]; unvaccinated, 92 [25.4%] vs 225 [29.5%], respectively). SARS-CoV-2 anti-N antibody detection was similar by COVID-19 vaccination status (eTable in Supplement 1). There was some vaccination status diversity within households (eFigure 1 in Supplement 1). Most participants who received a COVID-19 vaccine 12 months or less before the primary case participant received the 2023-2024 COVID-19 vaccine (264 [90.4%]) (Figure 2). Among primary case participants, the specific lineage of their SARS-CoV-2 infection varied by symptom onset date (ie, time of enrollment); similarly, their most recently received COVID-19 vaccine also varied over time (eFigure 2 in Supplement 1). Because of rolling enrollment over time, primary case participants who received their most recent COVID-19 vaccine 6 months or less before onset were distributed throughout the enrollment period.

Of the 763 included household contacts, 476 had SARS-CoV-2 infection detected during follow-up for an overall secondary infection risk of 62.4% (95% CI, 58.7% to 65.5%). All primary case participants reported symptoms, and 365 household contacts with SARS-CoV-2 infection (76.7%) reported symptoms. The median time from primary case participant symptom onset to enrollment was 4 days (IQR, 3-5 days) and was similar by vaccination status (eTable in Supplement 1). The median time among household contacts with SARS-CoV-2 infection from symptom onset to enrollment was 0 days (IQR, −1 to 1 day) and was similar by vaccination status.

### COVID-19 VE

**Figure 3.**
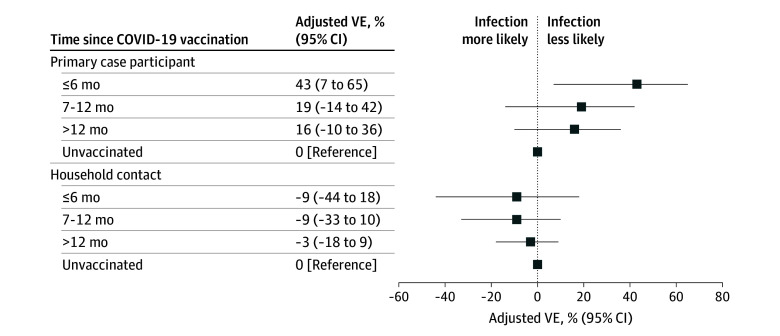
Forest Plot of COVID-19 Vaccine Effectiveness (VE) Against SARS-CoV-2 Infection, by Household Contact Vaccination Status Vaccine effectiveness was calculated as 1 minus the adjusted relative risk (Table 2). The adjusted relative risk was estimated using a modified Poisson regression model accounting for clustering at the household level and adjusting for the primary case participant COVID-19 vaccination status, household contact COVID-19 vaccination status, primary case participant age, household contact age, enrollment state, total number of people in the household, and enrollment period. COVID-19 vaccination status was defined according to months from the most recent dose to primary case participant onset and grouped into the following categories: 6 months or less, 7 to 12 months, and more than 12 months. Participants who did not have at least 1 verified COVID-19 vaccine dose or self-reported at least 1 vaccine dose with a vaccination date and either a vaccine manufacturer or vaccination location were considered unvaccinated.

In the multivariable model, household contacts of primary case participants who were vaccinated 6 months or less before onset had a lower risk of infection compared with contacts whose primary case participant was unvaccinated (ARR, 0.57% [95% CI, 0.35-0.93]) (relative risk in Table 2; VE in Figure 3). Household contacts of a primary case participant who received a vaccination 7 to 12 months and more than 12 months prior showed no statistically significant difference in infection risk compared with contacts of unvaccinated primary case participants (7-12 months: ARR, 0.81 [95% CI, 0.58-1.14]; >12 months: ARR, 0.84 [95% CI, 0.64-1.10]). The VEi estimates were not statistically significant from 0 for household contacts vaccinated 6 months or less, 7 to 12 months, and more than 12 months prior to onset in the primary case participant compared with unvaccinated contacts (≤6 months: VEi, −9% [95% CI, −44% to 18%]; 7-12 months: VEi, −9% [95% CI, −33% to 10%]; >12 months: VEi, −3% [95% CI, −18% to 9%). The sensitivity analysis showed similar results (eFigure 3 in Supplement 1).

**Table 2.** Relative Risk of SARS-CoV-2 Infection by COVID-19 Vaccination Status and Other Participant and Household Characteristics

Characteristic	No. of contacts with infection/No. of contacts	SIR, % (95% CI)^a^	CRR (95% CI)^b^	ARR (95% CI)^c^
COVID-19 vaccination status of primary case participant^d^				
≤6 mo ago	22/55	40.0 (28.1-53.2)	0.56 (0.38-0.82)	0.57 (0.35-0.93)
7-12 mo ago	70/118	59.3 (50.3-67.8)	0.78 (0.63-0.95)	0.81 (0.58-1.14)
>12 mo ago	186/333	55.9 (50.5-61.1)	0.75 (0.64-0.87)	0.84 (0.64-1.10)
Unvaccinated	198/257	77.0 (71.5-81.8)	1 [Reference]	1 [Reference]
COVID-19 vaccination status of household contact^d^				
≤6 mo ago	33/69	47.8 (36.5-59.4)	0.83 (0.65-1.06)	1.09 (0.82-1.44)
7-12 mo ago	74/120	61.7 (52.7-69.9)	0.98 (0.83-1.16)	1.09 (0.90-1.33)
>12 mo ago	219/349	62.8 (57.6-67.7)	0.97 (0.85-1.10)	1.03 (0.91-1.18)
Unvaccinated	150/225	66.7 (60.3-72.5)	1 [Reference]	1 [Reference]
Age of primary case participant, y				
0-4	162/198	81.8 (75.8-86.6)	1.40 (1.20-1.65)	1.16 (0.87-1.56)
5-17	66/129	51.2 (42.6-59.6)	0.91 (0.72-1.16)	0.87 (0.68-1.11)
18-49	171/301	56.8 (51.2-62.3)	1 [Reference]	1 [Reference]
≥50	77/135	57.0 (48.6-65.1)	1.02 (0.83-1.24)	1.04 (0.83-1.31)
Age of household contact, y				
0-4	51/66	77.3 (65.7-85.8)	1.14 (0.99-1.31)	1.19 (1.02-1.39)
5-17	115/200	57.5 (50.6-64.1)	1.08 (0.94-1.23)	1.08 (0.95-1.24)
18-49	212/344	61.6 (56.4-66.6)	1 [Reference]	1 [Reference]
≥50	98/153	64.1 (56.2-71.2)	1.09 (0.94-1.25)	1.16 (1.01-1.34)
Enrollment state				
New York	175/227	77.1 (71.2-82.1)	1.34 (1.16-1.56)	1.16 (0.97-1.39)
Tennessee	223/406	54.9 (50.1-59.7)	1 [Reference]	1 [Reference]
Washington	78/130	60.0 (51.4-68.0)	1.06 (0.86-1.30)	1.12 (0.91-1.39)
No. of people in household				
<4	183/308	59.4 (53.8-64.8)	1 [Reference]	1 [Reference]
≥4	293/455	64.4 (59.9-68.7)	1.10 (0.95-1.27)	1.02 (0.87-1.20)
Enrollment timing				
January 1 to June 30, 2024	202/345	58.6 (53.3-63.6)	1 [Reference]	1 [Reference]
July 1, 2024, to January 31, 2025	274/418	65.6 (60.9-69.9)	1.09 (0.94-1.26)	1.07 (0.92-1.23)

Abbreviations: ARR, adjusted relative risk; CRR, crude relative risk; SIR, secondary infection risk.

^a^ Calculated as the number of household contacts who had at least 1 nasal swab test positive for SARS-CoV-2 divided by all included household contacts. Estimated using Agresti-Coull binomial intervals.

^b^ Estimated for each characteristic using a modified Poisson regression model accounting for clustering at the household level and the characteristic as the only estimator in the model.

^c^ Estimated using a modified Poisson regression model accounting for clustering at the household level and adjusting for primary case participant COVID-19 vaccination status, household contact COVID-19 vaccination status, primary case participant age, household contact age, enrollment state, total number of people in the household, and enrollment period.

^d^ Defined according to months from the most recent dose to the primary case participant’s symptom onset and grouped into the following categories: 6 months or less, 7 to 12 months, and more than 12 months. Participants who did not have at least 1 verified vaccine dose or self-reported at least 1 vaccine dose with a vaccination date and either a vaccine manufacturer or vaccination location were considered unvaccinated.

## Discussion

This cohort study found that individuals vaccinated against COVID-19 within 6 months of illness onset were significantly less likely to transmit SARS-CoV-2 to their household contacts compared with those who were unvaccinated (VEt, 43% [95% CI, 7%-65%). This finding illustrates that recent COVID-19 vaccination may have indirect benefits by decreasing transmission to others. The transmission risk when primary case participants were last vaccinated between 7 and 12 months and more than 12 months was lower but not statistically significant compared with transmission from unvaccinated primary case participants. This trend suggests that COVID-19 vaccine protection against transmission wanes over time, similar to studies that considered other VE end points, including protection against severe illness.^6^ In this study conducted almost 4 years after the pandemic started, there was no statistically significant difference in risk of becoming infected based on the household contact’s own vaccination status. Together, these results suggest that in high-exposure settings, such as households, one possible way to protect other household members is to get a COVID-19 vaccine. These results may be relevant to other high-exposure settings such as health care facilities and congregate work or living settings.

While resource intensive, household transmission studies are unique in their ability to study factors associated with infection and transmission.^5^ Household studies performed during 2022 to 2023 found that primary case participant vaccination was associated with a reduced odds of transmission to household contacts^7^ and that household contacts with hybrid immunity (immunity from both vaccination and prior infection) had an associated reduced risk of being infected compared with those with no prior immunity.^4^ At the time that these studies were performed, a large, nationwide seroprevalence study estimated that population infection–induced immunity was nearly 90% among adults.^8^ Since, the level of population immunity to SARS-CoV-2 has increased as more and more of the population have had at least 1 prior infection with SARS-CoV-2. While this analysis was not designed to investigate the mechanism by which COVID-19 vaccination reduces transmission, prior immunity with boosting from recent COVID-19 vaccination may result in more efficient neutralization of SARS-CoV-2, thus decreasing infectiousness.^9^

### Limitations

Our study had several limitations. First, study participants may not be representative of the US population for several reasons, including that household recruitment required health care seeking and testing for SARS-CoV-2 soon after symptom onset. Second, secondary infection risk estimates did not account for multiple chains of transmission and ongoing SARS-CoV-2 infection risk from outside the household. Third, there was a small chance that the primary case participant identified during study eligibility screening and analysis of daily diary responses was not the first person with SARS-CoV-2 infection in the household if not all household members enrolled or the first person with SARS-CoV-2 was asymptomatic and never sought testing. Fourth, while the multivariable model accounted for within-household clustering, it did not address potential bias due to unmeasured factors and behaviors that could result in residual confounding and influence VE estimates. Fifth, sample size may have reduced the power to detect small effect sizes of VEi, leading to inconclusive results. Large cohort studies have shown conclusive VEi.^10,11^ Sixth, confirmed previous SARS-CoV-2 infection among primary case participants and household contacts was not included in estimating VE, which could bias estimates toward lower VE.^4^ However, because the US population had high seroprevalence of infection-induced SARS-CoV-2 immunity,^8^ these VE estimates may be interpreted as the incremental benefit of COVID-19 vaccination. Finally, household contact patterns between individuals are typically more frequent and physically closer, representing relatively high exposure compared with other nonhousehold settings, so these findings may not apply to low-exposure settings.

## Conclusions

This cohort study found that at a time when the US population had high levels of SARS-CoV-2 immunity, recent COVID-19 vaccination was associated with a decreased risk of SARS-CoV-2 transmission to others by nearly one-half. While the individual benefit of COVID-19 vaccination may vary by age and chronic medical conditions,^12,13^ COVID-19 vaccination may have indirect benefits by decreasing transmission and thus reducing overall exposure to SARS-CoV-2.^14^

## Notes

Supplement 1.
eMethods.

eReferences.
**eFigure 1.** Distribution of Household Contacts by Their Own Vaccination Status and Their Primary Case Participant Vaccination Status **eFigure 2.** Frequency of SARS-CoV-2 PAGNO Lineage, Most Recent COVID-19 Season Vaccine Received, and Time Since the Most Recent COVID-19 Vaccine by Symptom Onset Date Among Primary Case Participants **eFigure 3.** Relative Risk of Secondary COVID-19 Infection and COVID-19 Vaccine Effectiveness Against SARS-CoV-2 Transmission (Primary Case Participant Vaccination Status) and Infection (Household Contact Vaccination Status) Among Households With the Same Vaccination Status, New York, Tennessee, and Washington, January 2024 to January 2025 **eTable.** Indicator of Recent Prior SARS-CoV-2 Infection by Participant Type and Recent COVID-19 Vaccination Status, New York, Tennessee, and Washington, January 2024 to January 2025

Supplement 2.
Data Sharing Statement
