# Error in Figure

**DOI:** 10.1001/jamanetworkopen.2026.23403

**Published:** 2026-06-18

In the Original Investigation titled “Recent COVID-19 Vaccination and Risk of SARS-CoV-2 Transmission,”^1^ published May 15, 2026, Figure 3 contained an error in the vaccine effectiveness 95% CI for primary case participants at 6 months or less since COVID-19 vaccination. The 95% CI should have appeared as 7% to 65%. This article has been corrected.^1^
