# Supplementary material for: Recent COVID-19 Vaccination and Risk of SARS-CoV-2 Transmission
Source: JAMA Netw Open. 2026 May 15;9(5):e2612609. doi: 10.1001/jamanetworkopen.2026.12609 (PMC13179551; doi:10.1001/jamanetworkopen.2026.12609)
Supplement: Supplement 1. — eMethods. eReferences. eFigure 1. Distribution of Household Contacts by Their Own Vaccination Status and Their Primary Case Participant Vaccination Status eFigure 2. Frequency of SARS-CoV-2 PAGNO Lineage, Most Recent COVID-19 Season Vaccine Received, and Time Since the Most Recent COVID-19 Vaccine by Symptom Onset Date Among Primary Case Participants eFigure 3. Relative Risk of Secondary COVID-19 Infection and COVID-19 Vaccine Effectiveness Against SARS-CoV-2 Transmission (Primary Case Participant Vaccination Status) and Infection (Household Contact Vaccination Status) Among Households With the Same Vaccination Status, New York, Tennessee, and Washington, January 2024 to January 2025 eTable. Indicator of Recent Prior SARS-CoV-2 Infection by Participant Type and Recent COVID-19 Vaccination Status, New York, Tennessee, and Washington, January 2024 to January 2025 [file jamanetwopen-e2612609-s001.pdf]

Supplemental Online Content

Benist SC, Smith-Jeffcoat SE, Talbot HK, et al. Recent COVID-19 vaccination and risk of SARS-CoV-2 transmission. *JAMA Netw Open*. 2026;9(5):e2612609.
doi:10.1001/jamanetworkopen.2026.12609

eMethods

eReferences

eFigure 1. Distribution of Household Contacts by Their Own Vaccination Status and Their Primary Case Participant Vaccination Status

eFigure 2. Frequency of SARS-CoV-2 PAGNO Lineage, Most Recent COVID-19 Season Vaccine Received, and Time Since the Most Recent COVID-19 Vaccine by Symptom Onset Date Among Primary Case Participants

eFigure 3. Relative Risk of Secondary COVID-19 Infection and COVID-19 Vaccine Effectiveness Against SARS-CoV-2 Transmission (Primary Case Participant Vaccination Status) and Infection (Household Contact Vaccination Status) Among Households With the Same Vaccination Status, New York, Tennessee, and Washington, January 2024 to January 2025

eTable. Indicator of Recent Prior SARS-CoV-2 Infection by Participant Type and Recent COVID-19 Vaccination Status, New York, Tennessee, and Washington, January 2024 to January 2025

This supplemental material has been provided by the authors to give readers additional information about their work.

© 2026 Benist SC et al. *JAMA Network Open*.

eMethods

Study recruitment and enrollment

Primary case participants who tested positive for SARS-CoV-2 from the Respiratory Infection: Gauge of Household Transmission (RIGHT) study were identified through outpatient clinics, urgent care clinics, emergency departments, other prospective studies testing for SARS-CoV-2, or community outreach flyers. Primary case participants were enrolled within 6 days of illness onset which was defined as the first day of symptoms or, if asymptomatic, first positive test. Household contacts were eligible for the RIGHT study if they slept in the same household as the primary case participant ≥1 nights during the 7 days prior to primary case participant onset and planned to remain in household for 10 days after enrollment.

Collection of Race and Ethnicity

Self-reported race and ethnicity were collected at baseline to help describe the participants included in this study. For race, participants could select all that apply among the following options: American Indian or Alaska Native, Asian, Black or African American, Middle Eastern or North African, Native Hawaiian or Pacific Islander, White, Other race, Participant does not know race, Participant prefers not to answer about race. For Ethnicity, participants selected one of the following options: Hispanic, Latino(a), or Spanish origin, Not of Hispanic, Latino(a), or Spanish origin, I don't know, Prefer not to answer.

Collection of Gender and Reporting of Sex

Participants self-reported gender identity at baseline by selecting from the following options: Female, Male, Non-binary/Transgender, Other, or Prefer not to answer. In early February 2025, after all baseline data collection was complete, investigators were required to submit an IRB amendment to clarify that the collected data will be reported as Sex with only two options: Female or Male. Participants who self-reported gender as Female were categorized as Female sex, those who self-reported gender as Male were categorized as Male sex, and those who identified as "non-binary/transgender" or "other" or responded "preferred not to answer", were categorized as Unknown sex.

COVID-19 vaccination verification

Participants self-reported COVID-19 vaccination history at enrollment. Study staff verified self-reported vaccination history using state immunization registries, electronic medical records, pharmacy records, and provider records. COVID-19 vaccination status for this analysis was based on verified COVID-19 vaccine doses or plausible self-reported doses. Plausible self-reported doses were those that study staff were unable to verify, but the participant provided both a date of vaccination and at least one of vaccine manufacturer or location of vaccination dose receipt.

Blood collection and testing

Participants (or participant's guardian) collected baseline capillary whole blood sample using a Tasso+ (Tasso, Inc) collection kit. For one household, study staff collected whole blood sample through a standard, venipuncture blood draw. SARS-CoV-2 anti-nucleocapsid antibodies detection was performed using Meso Scale Discovery (MSD) V-plex SARS-CoV-2 panel 38 (IgG) Kit. Anti-nucleocapsid antibodies in samples collected <7 days after primary case onset were considered indicators of recent past infection that occurred prior to primary case onset. Previous studies illustrated IgG anti-N antibodies are present at least 6 months after acute SARS-CoV-2 infection.

© 2026 Benist SC et al. *JAMA Network Open*.

Nasal swab collection and testing

Nasal swabs were self-collected at home by enrolled participants and stored in Hologic transport media at room temperature. Study staff transported nasal swabs from the households every few days for transport to each site's local lab before final transport to the central testing laboratory at Vanderbilt University Medical Center. Nasal swabs were tested for SARS-CoV-2 via the Hologic Panther Fusion SARS-CoV-2 assay. Reverse transcription-polymerase chain reaction (RT-PCR) results were interpreted as categorically positive/negative according to the FDA-authorized parameters of the assay, as utilized for in vitro diagnostic purposes.

An aliquot of PCR-positive nasal swabs with an RT-PCR Ct value ≤32 was sent to the Lauring Laboratory at University of Michigan. Aliquots underwent viral whole genome sequencing using the ARTIC Network protocol on an Oxford Nanopore Technologies GridION instrument. SARS-CoV-2 lineages were assigned using the PANGO (phylogenetic assignment of named global outbreak) lineages nomenclatures. Only SARS-CoV-2 sequences that passed quality control rules were included in analyses.

Secondary infection risk

Secondary SARS-CoV-2 infection risk (SIR) was calculated as the number of household contacts positive for SARS-CoV-2 divided by all included household contacts. SIR 95% confidence intervals (CI) were estimated using Agresti-Coull binomial intervals.[1]

Modified Poisson model using GEE to estimate adjusted risk ratio

A modified Poisson model using generalized estimating equations with household clustering was used to estimate the relative risk of secondary infection and the COVID-19 vaccine effectiveness against infection and against transmission. While logistic regression models are typically used to estimate odds ratios of binary outcomes such as vaccine effectiveness, a modified or robust Poisson model can be used for common outcomes such as secondary infections of SARS-CoV-2 after household exposure.[2-4] Previous household studies estimating secondary infection risk of influenza and SARS-CoV-2 have used similar methods.[5,6]

Variables considered for the fully adjusted model were those that might confound vaccination and secondary infection risk using directed acyclic graph. These potential confounders included primary case participant age, household contact age, enrollment state, number of people in household, enrollment period, SARS-CoV-2 anti-N antibody detection status of primary case participant, and SARS-CoV-2 anti-N antibody detection status of household contact. Multicollinearity was assessed in the fully adjusted model, and variables with high variance inflation factors were removed from the final model. The removed variables included SARS-CoV-2 anti-N antibody detection status of primary case participant and household contact. Removing these variables did not affect the vaccination risk ratio point estimates, indicating that these measures of prior infection were not confounders in the multivariable model.

The final adjusted model included primary case participant vaccination status, household contact vaccination status, primary case participant age, household contact age, enrollment state, number of people in household, and enrollment period. Analyses were conducted in R (version 4.5.0; Posit) using geeglm in the geepack package.

© 2026 Benist SC et al. *JAMA Network Open*.

Sensitivity analysis among households with same vaccination status

A sensitivity analysis was preformed among households where all members had the same vaccination status. The final adjusted model was used to estimate vaccination effectiveness against transmission and infection.

Terminology on vaccine effectiveness

Vaccine effectiveness against transmission refers to how vaccination can impact the risk of infecting another person if someone is infected with a virus. VE against infectiousness is another term for this estimate.[7] Vaccine effectiveness against infection refers to how vaccination can impact a person's risk of getting infected. VE against susceptibility or VE against symptomatic infection are other terms used in this effect.[7]

© 2026 Benist SC et al. *JAMA Network Open*.

eReferences

1. Agresti A, and Coull BA. Approximate is Better than “Exact” for Interval Estimation of Binomial Proportions. *The American Statistician*. 1998/05/01 1998;52(2):119-126. doi:10.1080/00031305.1998.10480550
2. Zou GY, Donner A. Extension of the modified Poisson regression model to prospective studies with correlated binary data. *Stat Methods Med Res*. 2013:661-70. vol. 6.
3. McNutt L-A, Wu C, Xue X, Hafner JP. Estimating the Relative Risk in Cohort Studies and Clinical Trials of Common Outcomes. *American Journal of Epidemiology*. 2003;157(10):940-943. doi:10.1093/aje/kwg074
4. Chen W, Qian L, Shi J, Franklin M. Comparing performance between log-binomial and robust Poisson regression models for estimating risk ratios under model misspecification. *BMC Medical Research Methodology*. 2018/06/22 2018;18(1):63. doi:10.1186/s12874-018-0519-5
5. Rolfes MA, Talbot HK, McLean HQ, et al. Household Transmission of Influenza A Viruses in 2021-2022. *JAMA*. 2023;329(6):482-489. doi:10.1001/jama.2023.0064
6. Rolfes MA, Talbot HK, Morrissey KG, et al. Reduced risk of SARS-CoV-2 infection among household contacts with recent vaccination and past COVID-19 infection: results from 2 multisite case-ascertained household transmission studies. *American Journal of Epidemiology*. 2024;194(6):1603-1610. doi:10.1093/aje/kwae334
7. Halloran ME, Struchiner CJ, Longini IM, Jr. Study designs for evaluating different efficacy and effectiveness aspects of vaccines. *Am J Epidemiol*. Nov 15 1997;146(10):789-803. doi:10.1093/oxfordjournals.aje.a009196

© 2026 Benist SC et al. *JAMA Network Open*.

eFigure 1. Distribution of Household Contacts by Their Own Vaccination Status and Their Primary Case Participant Vaccination Status(n = 763)

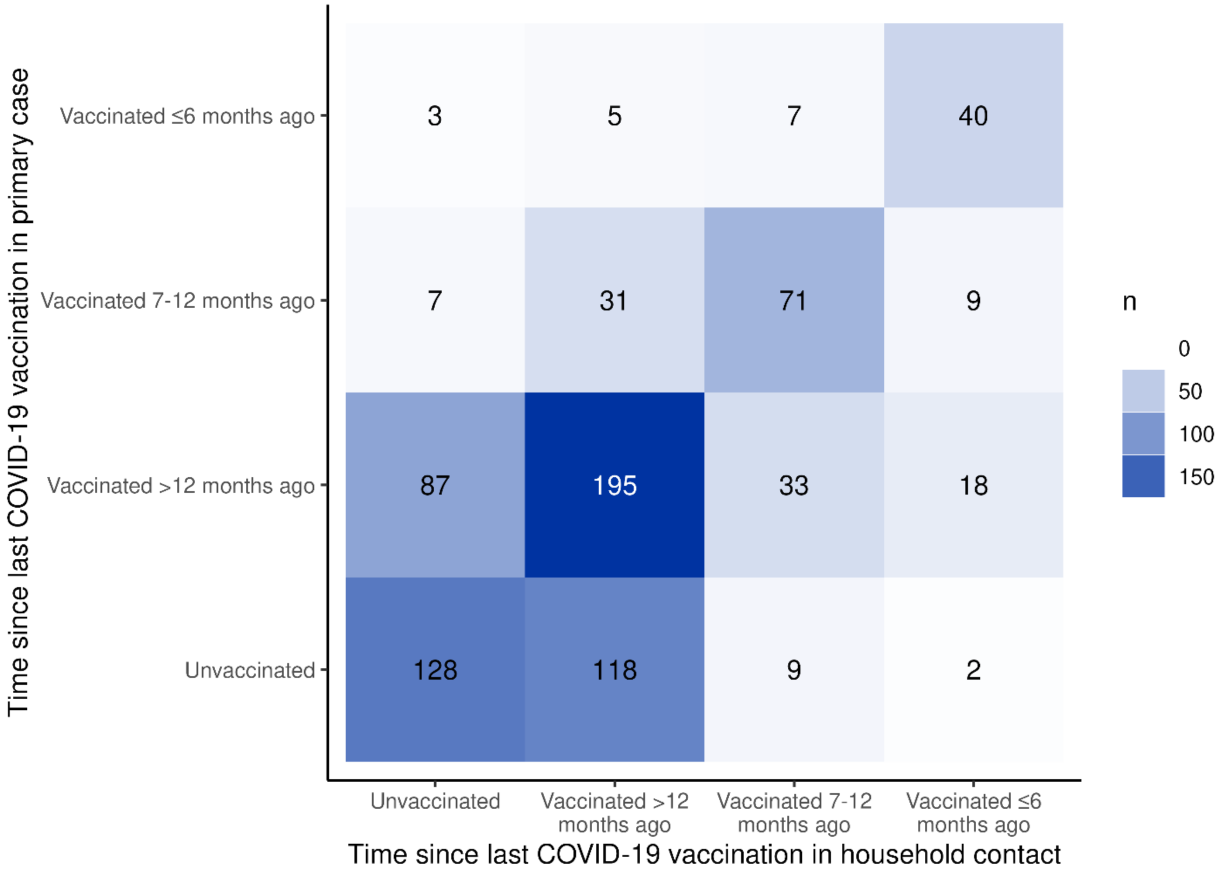

© 2026 Benist SC et al. *JAMA Network Open*.

eFigure 2. Frequency of SARS-CoV-2 PANGO Lineage[a] (A), Most Recent COVID-19 Season Vaccine Received[b] (B), and Time Since the Most Recent COVID-19 Vaccine[c] (C) by Symptom Onset Date Among Primary Case Participants (n = 362)

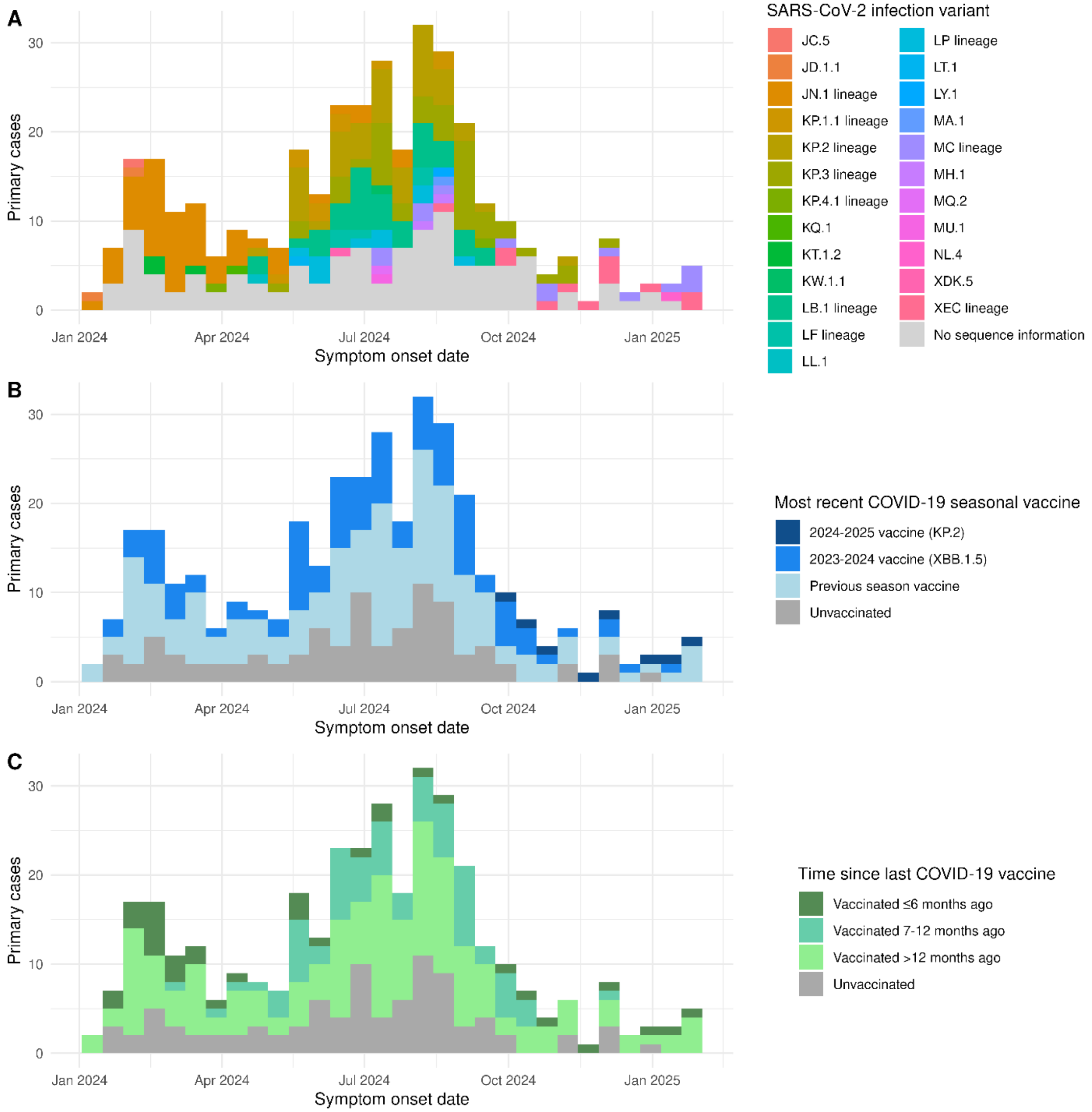

Footnote:

a) SARS-CoV-2 lineages were assigned using the Pangolin (phylogenetic assignment of named global outbreak lineages) nomenclatures. Only SARS-CoV-2 sequences that passed quality control rules were included in analyses. To display all lineages, direct sub lineages were grouped. Grouped lineages end in "lineage" (i.e. KP.1.1 grouped with KP.1.1.1 and KP.1.1.3 into "KP.1.1 lineage").

b) Vaccine season refers to the most recent COVID-19 vaccine received by the participant categorized as receiving a 2024-2025 COVID-19 vaccine, a 2023-2024 COVID-19 vaccine, or during a previous season prior to release of 2023-2024 COVID-19 vaccines. Participants who did not have ≥1 verified COVID-19 vaccine dose

© 2026 Benist SC et al. *JAMA Network Open*.

nor self-reported ≥1 vaccine dose with a vaccination date and either a vaccine manufacturer or vaccination location were considered unvaccinated.

c) COVID-19 vaccination timing was defined according to months from the most recent dose to primary case participant onset and grouped into the following categories: ≤6 months, 7-12 months, and >12 months. Participants who did not have ≥1 verified COVID-19 vaccine dose nor self-reported ≥1 vaccine dose with a vaccination date and either a vaccine manufacturer or vaccination location were considered unvaccinated.

© 2026 Benist SC et al. *JAMA Network Open*.

eFigure 3. Relative Risk of Secondary COVID-19 Infection and COVID-19 Vaccine Effectiveness[a] Against SARS-CoV-2 Transmission (Primary Case Participant Vaccination Status[b]) and Infection (Household Contact Vaccination Status[b]) Among Households With the Same Vaccination Status, New York, Tennessee, and Washington, January 2024 to January 2025

Characteristic	No. of contacts	No. of infected contacts	Crude infection risk (95% CI)	Crude RR (95% CI)	Adjusted RR (95% CI)	Adjusted VE (95% CI)
Vaccination status of household						
Vaccinated ≤6 months ago	34	13	38.2% (23.9%–55.0%)	0.69 (0.40–1.17)	0.51 (0.27–0.97)	49% (3%–73%)
Vaccinated 7-12 months ago	55	33	60.0% (46.8%–71.9%)	1.00 (0.69–1.45)	0.73 (0.44–1.22)	27% (-22%–56%)
Vaccinated >12 months ago	132	66	50.0% (41.6%–58.4%)	0.84 (0.59–1.20)	0.74 (0.45–1.21)	26% (-21%–55%)
Unvaccinated (Ref)	54	32	59.3% (46.0%–71.3%)	Reference		
Age of primary case, years						
0-4	35	21	60.0% (43.5%–74.5%)	1.18 (0.75–1.86)	1.12 (0.62–2.03)	
5-17	53	21	39.6% (27.6%–53.1%)	0.83 (0.53–1.30)	0.90 (0.58–1.38)	
18-49 (Ref)	105	50	47.6% (38.3%–57.1%)	Reference		
50+	82	52	63.4% (52.6%–73.0%)	1.25 (0.94–1.66)	1.25 (0.89–1.75)	
Age of household contact, years						
0-4	10	8	80.0% (47.9%–95.4%)	1.83 (1.32–2.53)	1.71 (1.19–2.45)	
5-17	70	34	48.6% (37.2%–60.0%)	1.21 (0.92–1.60)	1.16 (0.86–1.56)	
18-49 (Ref)	118	55	46.6% (37.9%–55.6%)	Reference		
50+	77	47	61.0% (49.9%–71.2%)	1.32 (1.01–1.72)	1.19 (0.88–1.63)	
Enrollment state						
New York	35	17	48.6% (33.0%–64.4%)	0.88 (0.55–1.41)	0.90 (0.56–1.45)	
Tennessee (Ref)	178	94	52.8% (45.5%–60.0%)	Reference		
Washington	62	33	53.2% (41.0%–65.1%)	0.99 (0.74–1.33)	1.13 (0.85–1.51)	
Number of people in household						
<4 people/house (Ref)	160	95	59.4% (51.6%–66.7%)	Reference		
≥4 people/house	115	49	42.6% (34.0%–51.7%)	0.77 (0.56–1.05)	0.81 (0.56–1.15)	
Enrollment timing						
January-June 2024 (Ref)	136	55	40.4% (32.6%–48.8%)	Reference		
July 2024-January 2025	139	89	64.0% (55.8%–71.5%)	1.45 (1.11–1.88)	1.44 (1.11–1.87)	

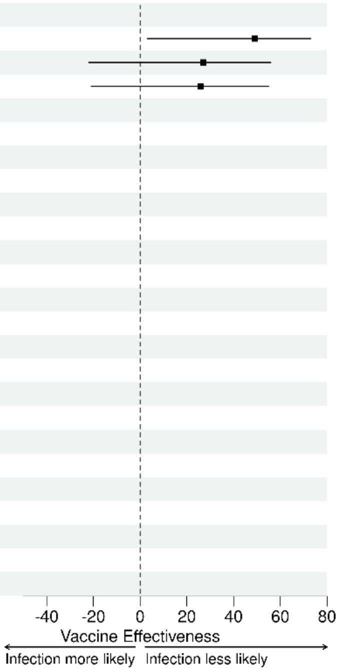

Footnotes:

Abbreviations: Ref=referent group

a) Vaccine effectiveness calculated as 1-adjusted risk ratio (aRR). aRR was estimated using a modified Poisson regression model accounting for clustering at the household level and adjusting for primary case participant COVID-19 vaccination status, household contact COVID-19 vaccination status, primary case participant age, household contact age, enrollment state, total number of people in the household, and enrollment period.

b) COVID-19 vaccination status was defined according to months from the most recent dose to primary case participant onset and grouped into the following categories: ≤6 months, 7-12 months, and >12 months. Participants who did not have ≥1 verified COVID-19 vaccine dose nor self-reported ≥1 vaccine dose with a vaccination date and either a vaccine manufacturer or vaccination location were considered unvaccinated.

© 2026 Benist SC et al. *JAMA Network Open*.

eTable. Indicator of Recent Prior SARS-CoV-2 Infection by Participant Type[a] and Recent COVID-19 Vaccination Status,[b] New York, Tennessee, and Washington, January 2024 to January 2025

	Primary case participants				Household contacts			
**Characteristic**	**Vaccinated ≤6 months ago** N = 35	**Vaccinated 7-12 months ago** N = 69	**Vaccinated >12 months ago** N = 166	**Unvaccinated** N = 92	**Vaccinated ≤6 months ago** N = 69	**Vaccinated 7-12 months ago** N = 120	**Vaccinated >12 months ago** N = 349	**Unvaccinated** N = 225
**Baseline SARS-CoV-2 anti-N detection**[c]								
Evidence of recent prior SCV2 infection	12 (34%)	29 (42%)	87 (52%)	27 (29%)	23 (33%)	43 (36%)	172 (49%)	92 (41%)
No evidence of recent prior SCV2 infection	16 (46%)	26 (38%)	30 (18%)	10 (11%)	22 (32%)	44 (37%)	86 (25%)	23 (10%)
No blood specimen collected <1 week	7 (20%)	14 (20%)	49 (30%)	55 (60%)	24 (35%)	33 (28%)	91 (26%)	110 (49%)
**Age at enrollment, years**								
0-4	2 (5.7%)	1 (1.4%)	1 (0.6%)	62 (67%)	5 (7.2%)	6 (5.0%)	5 (1.4%)	50 (22%)
5-17	2 (5.7%)	9 (13%)	22 (13%)	16 (17%)	10 (14%)	24 (20%)	73 (21%)	93 (41%)
18-49	10 (29%)	24 (35%)	96 (58%)	11 (12%)	24 (35%)	48 (40%)	198 (57%)	74 (33%)
50+	21 (60%)	35 (51%)	47 (28%)	3 (3.3%)	30 (43%)	42 (35%)	73 (21%)	8 (3.6%)
**Sex**								
Female	17 (49%)	30 (43%)	103 (62%)	49 (53%)	39 (57%)	64 (53%)	182 (52%)	114 (51%)
Male	18 (51%)	36 (52%)	60 (36%)	41 (45%)	28 (41%)	53 (44%)	158 (45%)	109 (48%)
Unknown/Missing	0 (0%)	3 (4.3%)	3 (1.8%)	2 (2.2%)	2 (2.9%)	3 (2.5%)	9 (2.6%)	2 (0.9%)
**Reported any symptoms**								
Yes	35 (100%)	69 (100%)	166 (100%)	92 (100%)	31 (45%)	64 (53%)	163 (47%)	107 (48%)
No					2 (2.9%)	10 (8.3%)	56 (16%)	43 (19%)
Not infected					36 (52%)	46 (38%)	130 (37%)	75 (33%)
**SARS-CoV-2-positive nasal swabs**								
Multiple positives	30 (86%)	66 (96%)	146 (88%)	81 (88%)	31 (94%)	63 (85%)	173 (79%)	128 (85%)
One positive	5 (14%)	3 (4.3%)	20 (12%)	11 (12%)	2 (6%)	11 (15%)	46 (21%)	22 (15%)
Not infected					36	46	130	75
**Days from symptom onset to enrollment**	4 (2, 5)	4 (2, 4)	4 (3, 5)	4 (3, 5)	0 (-1, 1)	0 (0, 2)	0 (-1, 1)	0 (-1, 1)
Did not report symptoms					19	26	104	70

Footnotes:

a) Participant type is defined as either being a primary case (i.e., the first confirmed case with earliest onset in the household) or a household contact;

© 2026 Benist SC et al. *JAMA Network Open*.

b) COVID-19 vaccination status was defined according to months from the most recent dose to primary case participant onset and grouped into the following categories: ≤6 months, 7-12 months, and >12 months. Participants who did not have ≥1 verified COVID-19 vaccine dose nor self-reported ≥1 vaccine dose with a vaccination date and either a vaccine manufacturer or vaccination location were considered unvaccinated;

c) Evidence of recent SARS-CoV-2 infection was defined as having detectable SARS-CoV-2 anti-nucleocapsid antibodies in a blood specimen collected within one week of primary case participant symptom onset. No evidence of recent SARS-CoV-2 infection was defined as not having detectable SARS-CoV-2 anti-N antibodies in a blood specimen collected within one week of primary case participant symptom onset.

© 2026 Benist SC et al. *JAMA Network Open*.
