# Supplementary material for: Recent COVID-19 Vaccination and Risk of SARS-CoV-2 Transmission
Source: JAMA Netw Open. 2026 May 15;9(5):e2612609. doi: 10.1001/jamanetworkopen.2026.12609 (PMC13179551; doi:10.1001/jamanetworkopen.2026.12609)
Supplement: Supplement 2. — Data Sharing Statement [file jamanetwopen-e2612609-s002.pdf]

Data Sharing Statement

Data
Data available: No

Additional Information
Explanation for why data not available: Data can be made available upon reasonable request to the corresponding author.
